# Membrane progesterone and oestrogen receptors modulate GABAergic transmission in the prefrontal cortex of prepubertal male, but not female, mice

**DOI:** 10.1113/EP092439

**Published:** 2025-05-01

**Authors:** Aitana Vázquez‐Sola, Hortensia Torres‐Torrelo, Josué García Yagüe

**Affiliations:** ^1^ Grupo de Neurofisiología Celular, Departamento de Ciencias Médicas Básicas, Facultad de Medicina, Instituto de Medicina Molecular Aplicada‐Nemesio Díez (IMMA‐ND) Universidad San Pablo‐CEU, CEU Universities, Urbanización Montepríncipe s/n Madrid Spain

**Keywords:** GABAergic transmission, G protein‐coupled oestrogen receptor, membrane progesterone receptor, prefrontal cortex, sex differences

## Abstract

The sex hormones progesterone (P) and oestrogen (E) reorganize GABAergic transmission in the prefrontal cortex (PFC) during the transition from childhood to adolescence, generating a new excitatory–inhibitory balance necessary for the computational capacity of the mature PFC. Little is known, however, about the hormone receptors involved or whether there are sex differences in the modulation of GABAergic transmission they exert. We hypothesize that P and E can rapidly (within minutes) modulate GABAergic currents through G protein‐coupled receptors, namely membrane P receptors (mPRs) and the G protein‐coupled E receptor (GPER), respectively, in PFC. First, we quantified the expression of P and E receptors in PFC using quantitative RT‐PCR. Secondly, we recorded synaptic (phasic) and extrasynaptic (tonic) GABAergic currents in basal conditions and in response to the activation of mPRs and GPER using patch‐clamp recordings in PFC neurons of prepubertal female and male mice. Expression levels of mPRs differed in the PFC of females and males, but no differences were found in the basal levels of phasic or tonic GABAergic currents between sexes. Interestingly, selective activation of mPRs increased tonic GABAergic transmission in males but not in females, and activation of GPER increased phasic GABAergic transmission only in males. We also demonstrated that GABAergic modulation exerted by mPRs and GPER was dependent on protein kinase A and C. This study sheds light on new mechanisms by which P and E can rapidly modulate GABAergic transmission in PFC neurons through the activation of mPRs and GPER.

## Abstract (highlights)

- **What is the central question of this study?**
  What is the role of membrane progesterone receptors and the G protein‐coupled oestrogen receptor as modulators of GABAergic transmission in the prefrontal cortex (PFC) of prepubertal mice and the possible sexual differences?
- **What is the main finding and its importance?**
  Activation of membrane progesterone receptors increases GABA_A_ receptor‐mediated tonic current in PFC pyramidal cells in male but not female mice. G protein‐coupled oestrogen receptor activation increases GABA_A_ receptor‐mediated phasic current in PFC pyramidal cells in male but not female mice. This study shows new mechanisms by which progesterone and oestrogen modulate GABAergic transmission in PFC neurons.

## 1 INTRODUCTION

The sex hormones progesterone (P) and oestrogen (E) participate in the structural and functional remodelling of prefrontal cortex (PFC) during the peripubertal period, i.e., the transition from childhood to adolescence (Caballero et al., 2014; Chini & Hanganu‐Opatz, 2021; Delevich et al., 2021; García‐Segura, 2009; Schulz et al., 2016). Among the functional changes, P and E enhance GABAergic transmission, leading to a new excitatory–inhibitory balance necessary for the development of the cognitive abilities associated with the mature PFC (Caballero & Tseng, 2016; Caballero et al., 2021; Chini & Hanganu‐Opatz, 2021; Klune et al., 2021; Piekarski, Boivin et al., 2017; Piekarski, Johnson et al., 2017; Pöpplau et al., 2024). The types of P and E receptors involved in this modulation are not known. P and E mediate many of their actions by interacting with classical P and E receptors (PRs and ERs, respectively) that function largely as transcription factors. In addition to these genomic actions, P and E can also mediate fast (non‐genomic) cell responses through these classical receptors and by the activation of diverse G protein‐coupled receptors (GPCRs), including five different membrane P receptors (mPRs), namely α, β, γ, δ and ε; in addition to a G protein‐coupled E receptor (GPER) (Alexander et al., 2017; Arévalo et al., 2015; Fuentes & Silveyra et al., 2019; González et al., 2020).

Apart from the well‐established positive allosteric modulation exerted by diverse P metabolites on the GABA_A_ receptors (GABA_A_Rs) (Belelli et al., 2009, 2019), some steroids might also regulate the efficacy of GABAergic inhibition through metabotropic mechanisms that involve the activation of diverse cell signalling pathways (Brown et al., 2015; Maguire & Mody, 2007). There are two forms of GABA_A_R‐mediated inhibition: a classical phasic inhibition mediated by GABA_A_Rs located at the synapse, which generates fast inhibitory postsynaptic currents (IPSCs), and a tonic GABA_A_R‐mediated current, produced by extrasynaptic GABA_A_Rs that generate persistent or tonic current (Belelli et al., 2009; Farrant & Nusser, 2005). Parakala et al. (2019) found that activation of mPRs can specifically increase the tonic GABA_A_R‐mediated current in hippocampal neurons of adult male mice. There is no evidence regarding whether mPRs could also modulate tonic current in PFC neurons during the prepubertal stage or whether sex differences exist. In addition to P, E can also modulate GABAergic transmission differently in various brain regions, not including the PFC (Clemens et al., 2019; Hernández‐Vivanco et al., 2022; Mukherjee et al., 2017; Tian et al., 2013). Less is known, however, about the E receptor(s) and signalling pathways involved in this modulation, particularly in the case of GPER. In this regard, it has been described that GPER activation acutely increases the frequency of IPSCs in the amygdala of adult female mice (Tian et al., 2013). It remains to be determined whether GPER could also modulate fast inhibitory synaptic transmission onto PFC neurons during the prepubertal stage, whether there are sex differences, and the possible signalling pathways involved.

There are no previous studies that have characterized the expression of each P and E receptor in the PFC of prepubertal male and female mice and, more importantly, the role that mPRs and GPER might have as modulators of GABAergic transmission during this crucial stage of the PFC in both sexes. For these reasons, in the present work, we first characterized the expression of the mRNAs for every P and E receptor (i.e., PR, mPRs, ERα, Erβ and GPER) in PFC, and secondly, we investigated the role of mPRs and GPER in the modulation of the synaptic (phasic) and extrasynaptic (tonic) GABAergic transmission using patch‐clamp recordings in PFC slices from prepubertal female and male mice.

## 2 MATERIALS AND METHODS

### 2.1 Ethical approval

In the present study, we used wild‐type prepubertal male and female C57BL/6J mice provided by the animal facility of San Pablo CEU University (SVA‐CEU.USP, REGA ES 28022 0000015). All procedures were conducted in accordance with the European Union Directive (2010/63/EU Directive) and the Spanish legislation (R.D. 53/2013 BOE 34/11370‐421) on the protection of animals used for scientific purposes and approved by the Ethics Board of the San Pablo CEU University. Animals were housed in standard cages with a 12 h–12 h light–dark cycle, maintained at room temperature, and ad libitum access to food and water. For brain extraction, mice were deeply anaesthetized with isoflurane and decapitated.

### 2.2 RNA extraction, retrotranscription and quantitative PCR analysis

Young prepubertal (postnatal days 20–26) female and male mice were deeply anaesthetized with isoflurane and decapitated. Brains were rapidly removed. The PFC was isolated and homogenized using a TissueLyser II (Qiagen). RNA was extracted with the RNeasy Lipid Tissue kit (Qiagen). To eliminate genomic DNA contamination, samples were treated with DNase (Qiagen). Two micrograms of the extracted RNA from each sample was used to synthesize complementary DNA (cDNA) by retrotranscription (RT) with the High‐Capacity RNATo‐cDNA Kit (Applied Biosystems). The resulting cDNA was used as a template for quantitative PCR (qPCR) with the PowerUp SYBR Master Mix (Applied Biosystems). The qPCR consisted of 45 cycles and an annealing temperature of 65°C using a CFX96 thermal cycler (Bio‐Rad). Specific primers against each of the mRNAs for every receptor (i.e., PR, mPRs, ERα, ERβ and GPER) were designed using a primer‐designing tool (US National Institutes of Health) (Table S1). The expression levels of these receptors were normalized using the glyceraldehyde 3‐phosphate dehydrogenase (*GAPDH*) gene as a reference gene. Relative quantification of the expression of each receptor was carried out following $2^{-\Delta \Delta C_{T}}$ (Livak & Schmittgen, 2001).

### 2.3 Slice preparation and whole‐cell patch‐clamp recordings

Young prepubertal (postnatal days 18–29) male and female C57BL/6J mice were anaesthetized with isoflurane, decapitated and the brains were rapidly removed. Coronal PFC slices 300 µm thick were cut in continuously oxygenated (95% O_2_–5% CO_2_) ice‐cold artificial cerebrospinal fluid (aCSF), as previously described (Ting et al., 2018), containing the following (mM): 92 NMDG, 2.5 KCl, 1.2 NaH_2_PO_4_.2H_2_O, 30 NaHCO_3_, 20 HEPES, 25 glucose, 5 sodium ascorbate, 2 thiourea, 3 sodium pyruvate, 10 MgSO_4_.7H_2_O and 0.5 CaCl_2_.2H_2_O. The solution was titrated at pH 7.3–7.4 with hydrochloric acid. Brain slices were initially stored in this solution at 32°C–34°C in an oxygenated recovery chamber. Na^+^ was added at the indicated times according to the aforementioned protocol. Then, slices were stored for ≥1 h in a recovery chamber with oxygenated aCSF at room temperature containing the following (mM): 92 NaCl, 2.5 KCl, 1.2 NaH_2_PO_4_.2H_2_O, 30 NaHCO_3_, 20 HEPES, 25 glucose, 5 sodium ascorbate, 2 thiourea, 3 sodium pyruvate, 2 MgSO_4_.7H_2_O and 2 CaCl_2_.2H_2_O. The solution was titrated at pH 7.3–7.4 with NaOH. Subsequently, slices were incubated for 15 min at 32°C ± 1°C in aCSF containing the following (mM): 124 NaCl, 2.5 KCl, 1.2 NaH_2_PO_4_.2H_2_O, 24 NaHCO_3_, 5 HEPES, 12.5 glucose, 2 MgSO_4_.7H_2_O and 2 CaCl_2_.2H_2_O. During this incubation, brain slices were treated either with the drug vehicle DMSO or with the corresponding drug (from Tocris Bioscience): ORG OD 02‐2, a selective agonist of mPRs; G1, a selective GPER agonist; KT 5720, a protein kinase A (PKA) inhibitor; and GF 109203X, a protein kinase C (PKC) inhibitor. Finally, brain slices were transferred to a recording chamber continuously perfused (∼2 mL/min) with warmed (32°C ± 1°C) oxygenated recording aCSF of the above composition plus 3 mM kynurenic acid (KA). The KA was used to block ionotropic glutamate receptors and, therefore, isolate GABA_A_R‐mediated currents.

Whole‐cell patch‐clamp recordings were carried out on layer 2/3 pyramidal (PYR) cells recorded from the dorsomedial PFC area 24b (Figure 2a; Paxinos & Franklin, 2019) and visualized using a Leyca DM6000 FS microscope equipped with a ×40 immersion lens and a Hamamatsu video camera. Neurons were held at −70 mV using pipettes (resistance, 4–4.5 MΩ) containing the following (mM): 133 CsCl, 1 MgCl_2_, 0.1 EGTA, 10 HEPES, 4 NaCl, 2 Mg‐ATP and 0.3 Na‐GTP (pH adjusted to 7.2 with CsOH, 290 mOsmol/Kg). With this solution, the reversal potential of Cl was ∼0 mV; therefore, GABA_A_R‐mediated currents appeared inward. After obtaining whole‐cell recordings, cells were allowed to stabilize for 5 min before currents were recorded using an Axopatch 200B amplifier (Molecular Devices) controlled by pClamp v.11 software (Molecular Devices). Series resistance and whole‐cell capacitance were determined in response to 5 mV voltage steps. Data were discarded if series resistance increased by >30%. Experimental data were digitized at 20 kHz (Digidata 1440A; Molecular Devices), acquired and analysed using pClamp v.11 software (Molecular Devices). To determine the presence of tonic GABA_A_R‐mediated current, the GABA_A_R antagonist picrotoxin was focally applied to the slice. Picrotoxin blocks the chloride channel of GABA_A_Rs, preventing its activation in response to the binding of GABA. Additionally, picrotoxin also blocks the spontaneous opening of the channel. This is important because part of the tonic inhibition is maintained by ligand‐independent, spontaneous opening of GABA_A_Rs (Wlodarczyk et al., 2013). The application of picrotoxin blocked IPSCs and produced an outward shift of the baseline current at a holding potential of −70 mV. The height of this shift corresponds to the magnitude of the tonic current. The total tonic current magnitude was also normalized to the whole‐cell capacitance for each neuron. For the IPSCs analysis, populations of individual IPSCs in every cell were averaged as described previously (Cope et al., 2005). We measured the IPSCs frequency, peak amplitude, area (charge transfer), rise slope and decay slope and compared these values between groups.

### 2.4 Morphological characterization of the recorded neurons

To identify the recorded neuron morphologically, biocytin (2 mg/mL) was added to the patch‐clamp pipette solution. At the end of the recording, slices were fixed in 4% paraformaldehyde for 24 h, followed by three rinses in PBS. Then, endogenous peroxidase was inhibited with a 3% hydrogen peroxide solution. Sections were subsequently processed using the Vectastain ABC Standard Kit (Vector Laboratories), and the biocytin distribution was detected histochemically with 0.05% 3,3′‐diaminobenzidine tetrahydrochloride (DAB) and 0.01% hydrogen peroxide (Sigma‐Aldrich). Sections were then rinsed with PBS and mounted on gelatin‐coated slides and left to air dry. Finally, they were dehydrated and coverslipped using Eukitt (Sigma‐Aldrich). Brain slices were visualized with a digital microscope (Leica CTR6000), and images were captured with Leica LAS‐X software (Figure 2a).

### 2.5 Statistical analysis

All the graphs and statistical analyses were carried out using GraphPad Prism software v.9.5.1. To compare two groups (e.g., females vs. males or two experimental conditions in patch‐clamp recordings), Student's unpaired *t*‐test was conducted when values within each group were normally distributed and exhibited equal variance between groups. When the values did not follow a normal distribution, the Mann–Whitney *U*‐test was conducted, and in cases where there was no homogeneity of variances, Welch's *t*‐test was used. To compare multiple groups (e.g., relative expression of different receptors or multiple experimental conditions in patch‐clamp recordings), a one‐way ANOVA followed by Dunnett's *post hoc* test was used when the values ​​followed a normal distribution and there was homogeneity of variances between groups. When variances were unequal, Welch's ANOVA followed by Dunnett's *post hoc* test was used. Data are expressed as the mean ± SD. Statistical significance was established at *P* < 0.05 for all analyses. The number of cells and mice used in each experimental group is represented by *n* and *N*, respectively.

## 3 RESULTS

### 3.1 Expression levels of mPRs differ in the PFC of females and males

**FIGURE 1:**
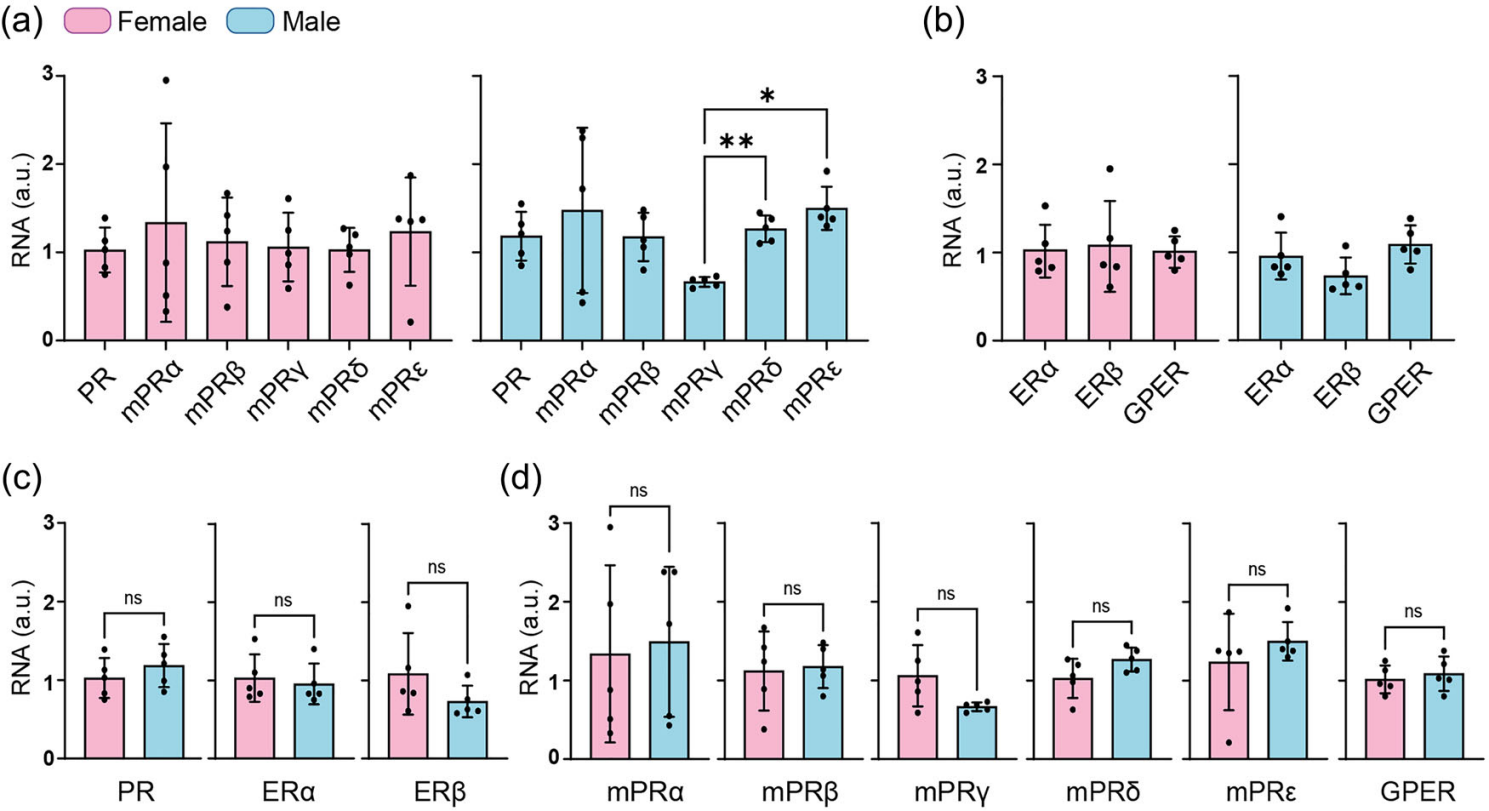
Relative expression of P and E receptor mRNAs in PFC of female (pink) and male (blue) mice. (a) Relative levels of PR and mPRs in females and males. (b) Relative levels of ERα, ERβ and GPER in females and males. (c) Comparison between females and males of relative levels of classical receptors (PR, ERα and ERβ). (d) Comparison between females and males of relative levels of GPCRs (mPRs and GPER). Data are shown as the mean ± SD; *P < 0.05, **P < 0.01; ns, no significant differences. (a) PR and mPRs in females (*P* = 0.974, Welch's ANOVA) and in males (*P* = < 0.0001, Welch's ANOVA with Dunnet's *post hoc* test (mPRγ vs. mPRδ, *P* = 0.004; mPRγ vs. mPRε, *P* = 0.014)). (b) ERα, ERβ and GPER in females (*P* = 0.951, one‐way ANOVA) and in males (*P* = 0.082, one‐way ANOVA). (c) Classical P and E receptors, female vs. male (PR, *P* = 0.373; ERα, *P* = 0.682; ERβ, *P* = 0.195, Student's unpaired *t*‐test). (d) mPRs and GPER, female vs. male (Student's unpaired *t*‐test: mPRα, *P* = 0.821; mPRβ, *P* = 0.832; mPRδ, *P* = 0.104; mPRε, *P* = 0.398; GPER, *P* = 0.583; Student's unpaired *t*‐test with Welch's correction: mPRγ, *P* = 0.086). Abbreviations: ERα, classical E receptor alpha; ERβ, classical E receptor beta; GPCRs, G protein‐coupled receptors; GPER, G protein‐coupled E receptor; mPRα, β, γ, δ and ε, membrane P receptor alpha, beta, gamma, delta and epsilon, respectively.

The qRT‐PCR results showed that PFC of prepubertal female and male mice expressed all the progesterone receptors, including PR and the five subtypes of mPRs, namely mPRα (*paqr7*), mPRβ (*paqr8*), mPRγ (*paqr5*), mPRδ (*paqr6*) and mPRε (*paqr9*) (Figure 1a). No differences were found in the relative expression of PR and mPRs in females (*P* = 0.974, Welch's ANOVA, *N* = 5; Figure 1a, left panel). Males, however, exhibited lower expression levels of mPRγ compared with mPRδ (*P* = 0.0039, Welch's ANOVA; *N* = 5) and mPRε (*P* = 0.014, Welch's ANOVA; *N* = 5; Figure 1a, right panel). Unlike P receptors, the expression levels of ERα, Erβ and GPER were similar within each sex (females, *P* = 0.951, one‐way ANOVA, *N* = 5; males, *P* = 0.082, one‐way ANOVA, *N* = 5; Figure 1b). When comparing each receptor between females and males, we did not observe sexual differences for any of the classical P and E receptors (PR, *P* = 0.373; ERα, *P* = 0.682; ERβ, *P* = 0.195; Student's unpaired *t*‐test, *N* = 5; Figure 1c) or for the membrane ones (mPRα, *P* = 0.821; mPRβ, *P* = 0.832; mPRδ, *P* = 0.104; mPRε, *P* = 0.398; GPER, *P* = 0.583; Student's unpaired *t*‐test, *N* = 5; mPRγ, *P* = 0.086, Student's unpaired *t*‐test with Welch's correction, *N* = 5; Figure 1d).

### 3.2 Tonic and phasic GABA_A_R‐mediated currents in PFC layer 2/3 PYR cells are similar in females and males

**FIGURE 2:**
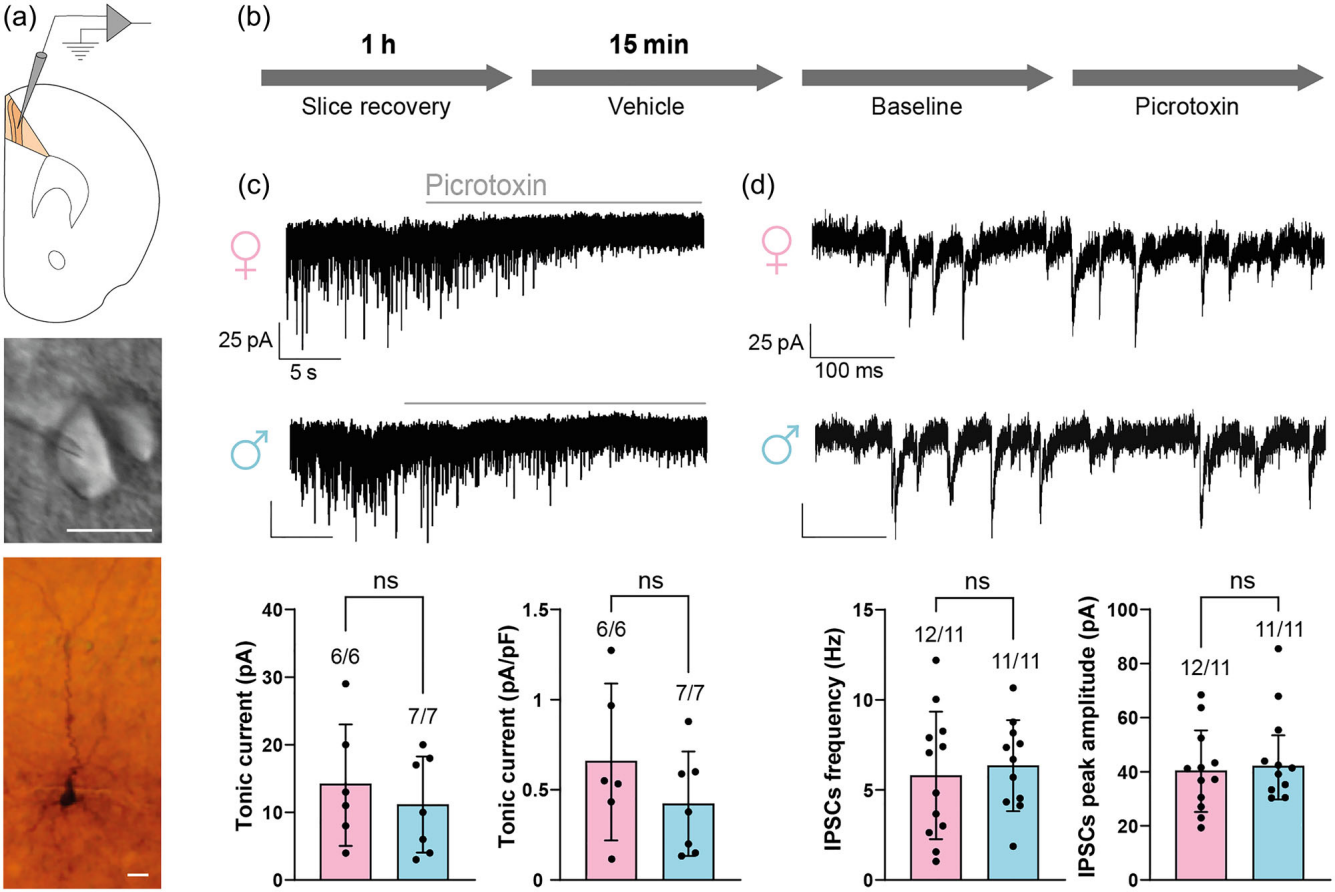
Basal levels of tonic and phasic GABA_A_R‐mediated currents in PFC layer 2/3 PYR cells in female (F) and male (M) mice. (a) Patch‐clamp recordings were carried out in layers 2/3 of area A24b of PFC (upper panel); an example of a layer 2/3 PYR cell during recording (middle panel) and its subsequent morphological characterization (bottom panel). (b) Protocol used for recordings in basal conditions. (c) Representative current traces from PFC slices treated with vehicle in females and males (upper panels) and average total (lower left panel) and normalized (lower right panel) tonic current. (d) Representative individual IPSCs (upper panels) and average frequency of IPSCs (lower left panel) and amplitude (lower right panel). Data are shown as the mean ± SD; ns, no significant differences. The figures above bars indicate the number of cells and animals (*n*/*N*, respectively). (c) Total tonic current, F vs. M: *P* = 0.512, Student's unpaired *t*‐test; normalized tonic current, F vs. M: *P* = 0.271, Student's unpaired *t*‐test. (d) Frequency of IPSCs, F vs. M: *P* = 0.677, Student's unpaired *t‐* test; (d) peak amplitude of IPSCs, F vs. M: *P* = 0.525, Mann–Whitney *U*‐test. Abbreviations: PFC, prefrontal cortex; PYR, pyramidal.

We measured the basal levels of both types of GABA_A_R‐mediated currents: the extrasynaptic (tonic) and synaptic (phasic) currents in layer 2/3 PYR cells (Figure 2c, d). Focal application of picrotoxin (100 µM) to layer 2/3 PYR cells not only blocked IPSCs but also produced an outward shift in the baseline current that revealed the presence of tonic current (Figure 2c). The magnitude of total tonic current was similar in females (14.2 ± 9 pA, *n* = 6, *N* = 6) and males (11.1 ± 7.1 pA, *n* = 7, *N* = 7; *P* = 0.512, Student's unpaired *t*‐test; Figure 2c). Similar results were observed when total tonic current was normalized to whole‐cell capacitance in females (0.7 ± 0.4 pA/pF, *n* = 5, *N* = 5) and males (0.4 ± 0.3 pA/pF; *P* = 0.271, Student's unpaired *t*‐test; Figure 2c). Phasic GABA_A_R‐mediated current was measured by analysing the IPSCs before picrotoxin application (Figure 2d). The frequency of IPSCs was similar between females (5.8 ± 3.5 Hz, *n* = 12, *N* = 11) and males (6.4 ± 2.5 Hz, *n* = 11, *N* = 11; *P* = 0.677, Student's unpaired *t*‐test), as was their amplitude in females (40.4 ± 15.2 pA, *n* = 12, *N* = 11) and males (46.1 ± 17.7 pA, *n* = 11, *N* = 11; *P* = 0.525, Mann–Whitney *U*‐test; Figure 2d). No differences were found between females and males in the IPSCs area, rise slope or decay slope (Table 1).

**TABLE 1:** Comparison of diverse parameters of IPSCs in prefrontal cortex layer 2/3 pyramidal cells between female and male mice in control conditions (CTRL) and in various experimental conditions (CTRL vs. ORG and CTRL vs. G1). Data show the mean ± SD; *n* and *N*, Number of cells and animals, respectively.

	CTRL	CTRL vs. ORG	CTRL vs. ORG	CTRL vs. G1	CTRL vs. G1
IPSC parameters	Females vs. males	Females	Males	Females	Males
Frequency, Hz	Females 5.8 ± 3.5	Control 5.8 ± 3.5	Control 6.3 ± 2.5	Control 5.8 ± 3.5	Control 6.3 ± 2.5
	Males 6.3 ± 2.5	ORG 6 ± 2.2	ORG 5.7 ± 3	G1 7.8 ± 2.7	G1 8.6 ± 2.3
	Females *n* = 12, *N* = 11	Control *n* = 12, *N* = 11	Control *n* = 11, *N* = 11	Control *n* = 12, *N* = 11	Control *n* = 11, *N* = 11
	Males *n* = 11, *N* = 11	ORG *n* = 13, *N* = 10	ORG *n* = 12, *N* = 10	G1 *n* = 16, *N* = 9	G1 *n* = 18, *N* = 10
	Student's unpaired *t*‐test	Student's unpaired *t*‐test	Student's unpaired *t*‐test	Student's unpaired *t*‐test	Student's unpaired *t*‐test
	*P* = 0.677	*P* = 0.835	*P* = 0.598	*P* = 0.096	*P* = 0.019
Peak amplitude, pA	Females 40.4 ± 15.2	Control 40.4 ± 15.2	Control 46.1 ± 17.7	Control 40.4 ± 15.2	Control 46.1 ± 17.7
	Males 46.1 ± 17.7	ORG 38.3 ± 10.6	ORG 41.3 ± 8.9	G1 43.3 ± 10.6	G1 39.3 ± 12.5
	Females *n* = 12, *N* = 11	Control *n* = 12, *N* = 11	Control *n* = 11, *N* = 11	Control *n* = 12, *N* = 11	Control *n* = 11, *N* = 11
	Males *n* = 11, *N* = 11	ORG *n* = 13, *N* = 10	ORG *n* = 12, *N* = 10	G1 *n* = 16, *N* = 9	G1 *n* = 18, *N* = 10
	Mann–Whitney *U*‐test	Mann–Whitney *U*‐test	Mann–Whitney *U*‐test	Student's unpaired *t*‐test	Mann–Whitney *U*‐test
	*P* = 0.525	*P* = 0.769	*P* = 0.88	*P* = 0.554	*P* = 0.412
Area, pA ms	Females 231 ± 95	Control 231 ± 94.9	Control 237 ± 88.7	Control 231 ± 94.9	Control 237 ± 88.7
	Males 237 ± 98	ORG 218 ± 60.4	ORG 260 ± 70	G1 209 ± 71.9	G1 196 ± 67.4
	Females *n* = 12, *N* = 11	Control *n* = 12, *N* = 11	Control *n* = 11, *N* = 11	Control *n* = 12, *N* = 11	Control *n* = 11, *N* = 11
	Males *n* = 11, *N* = 11	ORG *n* = 13, *N* = 10	ORG *n* = 12, *N* = 10	G1 *n* = 16, *N* = 9	G1 *n* = 18, *N* = 10
	Mann–Whitney *U*‐test	Mann–Whitney *U*‐test	Student's unpaired *t*‐test	Mann–Whitney *U*‐test	Student's unpaired *t*‐test
	*P* = 0.821	*P* = 0.936	*P* = 0.51	*P* = 0.397	*P* = 0.182
Rise slope, pA/ms	Females 35 ± 19	Control 34.8 ± 19.2	Control 44.9 ± 23	Control 34.8 ± 19.2	Control 44.9 ± 23
	Males 45 ± 23	ORG 28.6 ± 10.7	ORG 35.7 ± 17.9	G1 42.7 ± 16.3	G1 39.7 ± 24.1
	Females *n* = 12, *N* = 11	Control *n* = 12, *N* = 11	Control *n* = 11, *N* = 11	Control *n* = 12, *N* = 11	Control *n* = 11, *N* = 11
	Males *n* = 11, *N* = 11	ORG *n* = 13, *N* = 10	ORG *n* = 12, *N* = 10	G1 *n* = 16, *N* = 9	G1 *n* = 18, *N* = 10
	Student's unpaired *t*‐test	Student's unpaired *t*‐test	Student's unpaired *t*‐test	Student's unpaired *t*‐test	Mann–Whitney *U*‐test
	*P* = 0.278	*P* = 0.326	*P* = 0.291	*P* = 0.264	*P* = 0.363
Decay slope, pA/ms	Females 6 ± 1.8	Control 6 ± 1.8	Control 4.7 ± 1.4	Control 6 ± 1.8	Control 4.7 ± 1.4
	Males 4.7 ± 1.4	ORG 4.9 ± 1.6	ORG 5.1 ± 4.2	G1 7.3 ± 3.1	G1 5.6 ± 1.6
	Females *n* = 12, *N* = 11	Control *n* = 12, *N* = 11	Control *n* = 11, *N* = 11	Control *n* = 12, *N* = 11	Control *n* = 11, *N* = 11
	Males *n* = 11, *N* = 11	ORG *n* = 13, *N* = 10	ORG *n* = 12, *N* = 10	G1 *n* = 16, *N* = 9	G1 *n* = 18, *N* = 10
	Student's unpaired *t*‐test	Mann–Whitney *U*‐test	Mann–Whitney *U*‐test	Student's unpaired *t*‐test	Student's unpaired *t*‐test
	*P* = 0.078	*P* = 0.123	*P* = 0.487	*P* = 0.226	*P* = 0.165

### 3.3 Selective activation of mPRs produces a PKA‐ and PKC‐dependent increase in GABA_A_R‐mediated tonic current in PFC layer 2/3 PYR cells in males but not females

**FIGURE 3:**
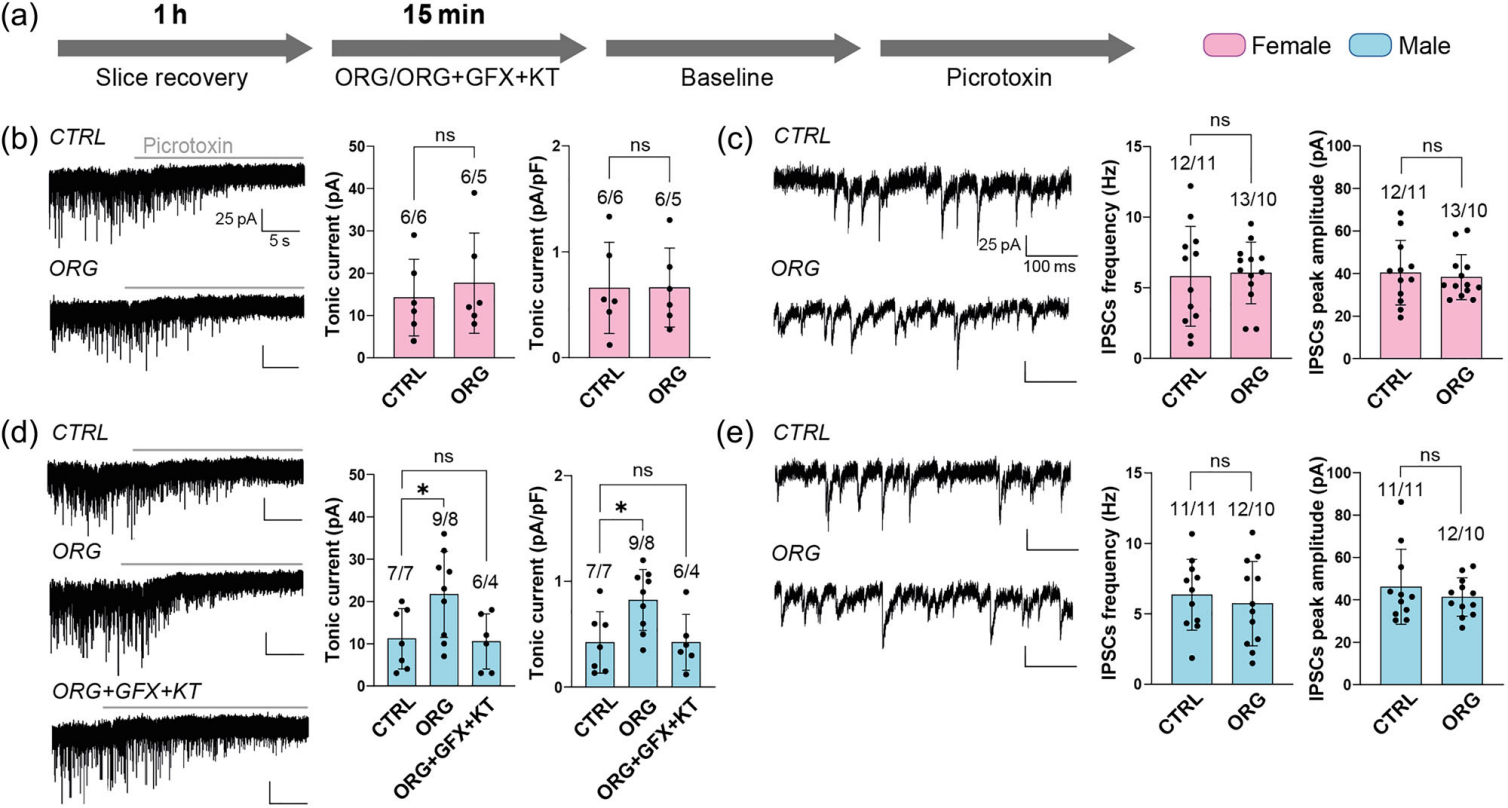
Tonic and phasic GABA_A_R‐mediated currents in PFC layer 2/3 PYR cells in female and male mice in response to selective activation of mPRs by ORG. (a) Protocol used for recordings in slices with vehicle (CTRL), 3 µM ORG and 3 µM ORG + 2 µM GFX + 0.1 µM KT. (b) Representative current traces with vehicle (CTRL) or ORG (left panels) and average total and normalized tonic current in females (right panels). (c) Representative individual IPSCs with vehicle (CTRL) or ORG (left panels) and average frequency and amplitude of IPSCs in females (right panels). (d) Representative current traces with vehicle (CTRL), ORG or ORG+GFX+KT (left panels) and average total and normalized tonic current in males (right panels). (e) Representative individual IPSCs with vehicle (CTRL) or ORG (left panels) and average frequency and amplitude of IPSCs in males (right panels). Data are shown as the mean ± SD; *P < 0.05; ns, no significant differences. The figures above bars indicate the number of cells and animals (*n*/*N*, respectively). (b) Total tonic current, CTRL vs. ORG in females: *P* = 0.577, Student's unpaired *t*‐test; normalized tonic current, CTRL vs. ORG in females: *P* = 0.976, Student's unpaired *t*‐test. (c) Frequency of IPSCs, CTRL vs. ORG in females: *P* = 0.385, Student's unpaired *t*‐test; peak amplitude of IPSCs, CTRL vs. ORG in females: *P* = 0.769, Mann–Whitney *U*‐test. (d) Total tonic current, CTRL vs. ORG vs. ORG+GFX+KT in males: *P* = 0.027, one‐way ANOVA (CTRL vs. ORG, *P* = 0.041; CTRL vs. ORG+GFX+KT, *P* = 0.986; Dunnett's *post hoc* test); normalized tonic current, CTRL vs. ORG vs. ORG+GFX+KT in males: *P* = 0.014, one‐way ANOVA (CTRL vs. ORG, *P* = 0.020; CTRL vs. ORG+GFX+KT, *P* > 0.999; Dunnett's *post hoc* test). (e) Frequency of IPSCs, CTRL vs. ORG in males: *P* = 0.598, Student's unpaired *t*‐test; peak amplitude of IPSCs, CTRL vs. ORG in males: *P* = 0.728, Mann–Whitney *U*‐test. Abbreviations: mPRs, membrane P receptors; PFC, prefrontal cortex; PYR, pyramidal.

Acute (15 min) treatment of PFC slices with the selective mPRs agonist ORG (ORG OD 02‐2, 3 µM) did not have any effect on the total tonic current in females (control 14.2 ± 9 pA, *n* = 6, *N* = 6; ORG 17.6 ± 11.8 pA, *n* = 6, *N* = 5; *P* = 0.577, Student's unpaired *t*‐test) or in the normalized values (control 0.7 ± 0.4 pA/pF, *n* = 6, *N* = 6; ORG 0.67 ± 0.4 pA/pF, *n* = 6, *N* = 5; *P* = 0.976, Student's unpaired *t*‐test; Figure 3b). Unlike females, ORG elicited a 94% increase in the total tonic current in males (control 11.1 ± 7.1 pA, *n* = 7, *N* = 7; ORG 21.7 ± 10.2 pA, *n* = 9, *N* = 8; *P* = 0.041, one‐way ANOVA with Dunnett's *post hoc* test; Figure 3d). A similar increase was observed when total tonic current was normalized to whole‐cell capacitance (control 0.4 ± 0.3 pA/pF, *n* = 7, *N* = 7; ORG 0.8 ± 0.3 pA/pF, *n* = 9, *N* = 8; *P* = 0.014, one‐way ANOVA with Dunnett's *post hoc* test; Figure 3d). This increase in tonic current was suppressed with the inhibition of PKA and PKC, as shown by the absolute values of tonic current (control 11.1 ± 7.1 pA, *n* = 7, *N* = 7; ORG+GFX+KT 10.5 ± 6.5 pA, *n* = 6, *N* = 4; *P *= 0.986, one‐way ANOVA with Dunnett's *post hoc* test) and the normalized ones (control 0.4 ± 0.3 pA/pF, *n* = 7, *N* = 7; ORG+GFX+KT 0.4 ± 0.3 pA/pF, *n* = 6, *N* = 4; *P* > 0.999, one‐way ANOVA with Dunnett's *post hoc* test; Figure 3d). Unlike its effect on tonic current in males, ORG did not affect the frequency, amplitude, area, rise slope or decay slope of IPSCs in either females or males (Figure 3c, e; Table 1).

### 3.4 Selective activation of GPER produces a PKA‐ and PKC‐dependent increase in the frequency of IPSCs in PFC layer 2/3 PYR cells in males but not females

**FIGURE 4:**
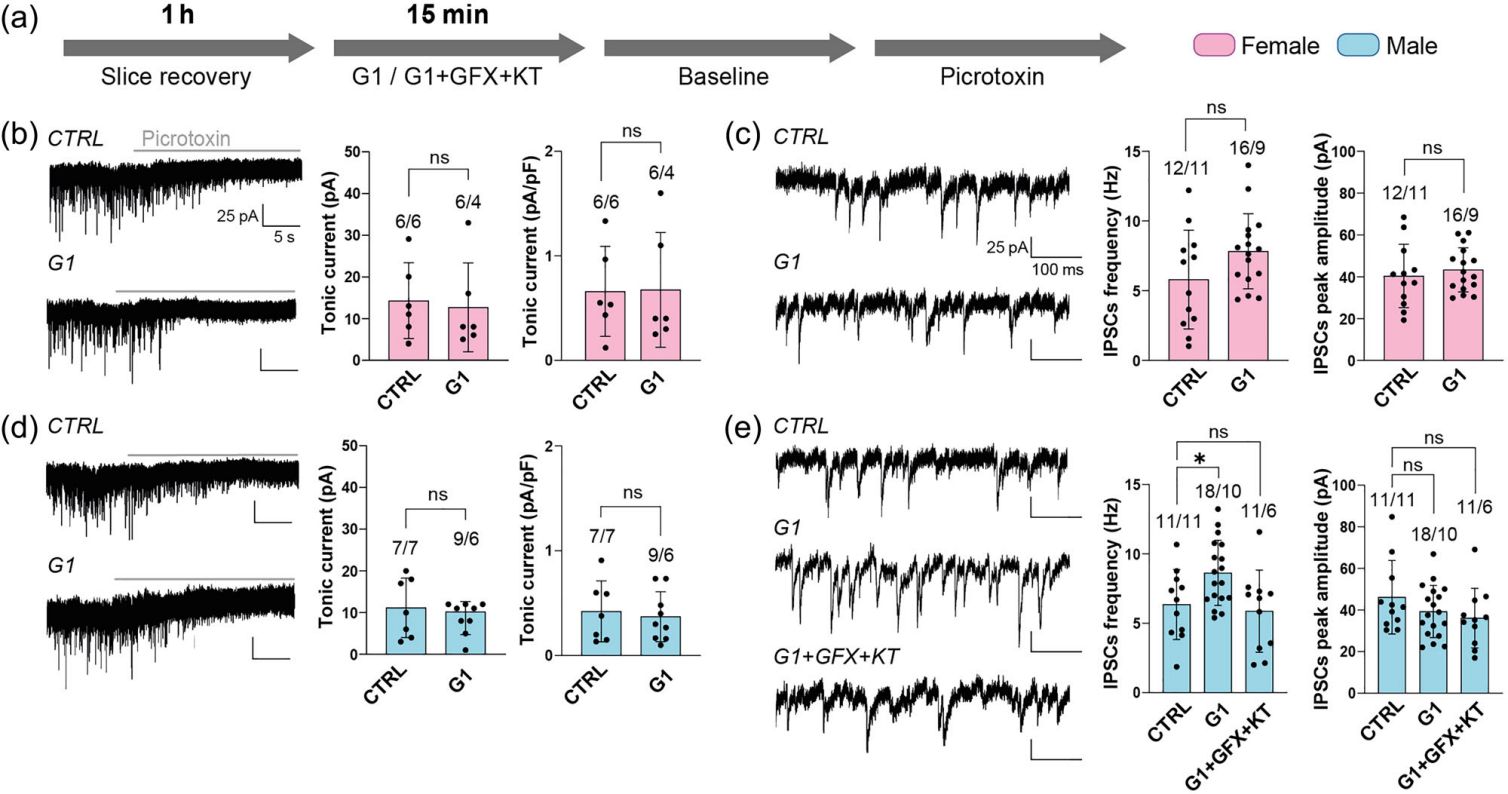
Tonic and phasic GABA_A_R‐mediated currents in PFC layer 2/3 PYR cells in female and male mice in response to selective activation of GPER by G1. (a) Protocol used for recordings in slices with vehicle (CTRL), 1 µM G1 or 1 µM G1 + 2 µM GFX + 0.1 µM KT. (b) Representative current traces with vehicle (CTRL) or G1 (left panels) and average total and normalized tonic current in females (right panels). (c) Representative individual IPSCs with vehicle (CTRL) or G1 (left panels) and average frequency and amplitude of IPSCs in females (right panels). (d) Representative current traces with vehicle (CTRL) or G1 (left panels) and average total and normalized tonic current in males (right panels). (e) Representative individual IPSCs with vehicle (CTRL), G1 and G1+GFX+KT (left panels), and average frequency and amplitude of IPSCs in males (right panels). Data are shown as the mean ± SD; *P < 0.05; ns, no significant differences. The figures above bars indicate the number of cells and animals (*n*/*N*, respectively). (b) Total tonic current, CTRL vs. G1 in females: *P* = 0.697, Mann–Whitney *U*‐test; normalized tonic current, CTRL vs. G1 in females: *P* = 0.667, Mann–Whitney *U*‐test. (c) Frequency of IPSCs, CTRL vs. G1 in females: *P* = 0.096, Student's unpaired *t*‐test; peak amplitude of IPSCs, CTRL vs. G1 in females: *P* = 0.554, Student's unpaired *t*‐test. (d) Total tonic current, CTRL vs. G1 in males: *P* = 0.737, Mann–Whitney *U*‐test; normalized tonic current, CTRL vs. G1 in males: *P* = 0.702, Student's unpaired *t* test. (e) Frequency of IPSCs, CTRL vs. G1 vs. G1+GFX+KT in males: *P* = 0.012, one‐way ANOVA (CTRL vs. G1, *P* = 0.048; CTRL vs. G1+GFX+KT, *P* = 0.788; Dunnett's *post hoc* test); peak amplitude of IPSCs, CTRL vs. G1 vs. G1+GFX+KT in males: *P* = 0.449, Kruskal–Wallis test. Abbreviations: GPER, G protein‐coupled E receptor; PFC, prefrontal cortex; PYR, pyramidal.

Acute (15 min) treatment with the selective GPER agonist G1 (1 µM) did not produce any change in the absolute values of tonic current (control 14.2 ± 9 pA, *n* = 6, *N* = 6; G1 12.7 ± 10.7 pA, *n* = 6, *N* = 4; *P* = 0.697, Mann–Whitney *U*‐test) or in the normalized ones (control 0.7 ± 0.4 pA/pF, *n* = 6, *N* = 6; G1 0.7 ± 0.5 pA/pF, *n* = 6, *N* = 4; *P* = 0.667, Mann–Whitney *U*‐test) in females (Figure 4b). G1 also did not affect the total tonic current (control 11.1 ± 7.1 pA, *n* = 7, *N* = 7; G1 8.9 ± 3.8 pA, *n* = 9, *N* = 6; *P* = 0.737, Mann–Whitney *U*‐test) or the normalized one (control 0.4 ± 0.3 pA/pF, *n* = 7, *N* = 7; G1 0.4 ± 0.2 pA/pF, *n* = 9, *N* = 6; *P* = 0.702, Student's unpaired *t*‐test) in males (Figure 4d). In contrast to the lack of effect on the tonic current, G1 did induce a 36% increase in the frequency of IPSCs in males (control 6.3 ± 2.5 Hz, *n* = 11, *N* = 11; G1 8.6 ± 2.3 Hz, *n* = 18, *N* = 10; *P* = 0.048 one‐way ANOVA with Dunnett's *post hoc* test; Figure 4e) but not in females (control 5.8 ± 3.5 Hz, *n* = 12, *N* = 11; G1 7.8 ± 2.7 Hz, *n* = 16, *N* = 9; *P* = 0.096, Student's unpaired *t*‐test; Figure 4c). This increase in the frequency of IPSCs in males was blocked by inhibition of PKA and PKC (control 6.3 ± 2.5, Hz, *n* = 11, *N* = 11; G1+GFX+KT 5.7 ± 3.1 Hz, *n* = 10, *N* = 6; *P* = 0.788, one‐way ANOVA with Dunnett's *post hoc* test; Figure 4e). G1 did not affect the amplitude, area, rise slope or decay slope of IPSCs in either females or males (Figure 4c, e; Table 1).

## 4 DISCUSSION

In this work, we have first characterized the expression levels of the mRNAs of each of the P and E receptors (i.e., mPRs, PR, ERα, ERβ and GPER) in PFC of prepubertal male and female mice and, secondly, we have demonstrated that mPRs and GPER can acutely modulate (within minutes) GABAergic transmission selectively in PFC of prepubertal male mice. More specifically, we show that selective activation of mPRs by ORG produces an increase in the tonic current in layer 2/3 PYR cells without altering the phasic current and, in contrast, selective activation of GPER by G1 promotes an increase in the frequency of the IPSCs without affecting the tonic current. We also demonstrate that GABAergic modulation exerted by mPRs and GPER is dependent on protein kinase A and C.

Regarding the expression of P and E receptors, our qPCR results show that PFC of prepubertal females and males expresses both the classical receptors ERα, ERβ and PR and the different subtypes of mPRs (α, β, γ, δ and ε) and GPER. Our data confirm and expand previous evidence on the presence of diverse P receptors in other species, brain regions, ages and sexes, including the expression of PR in the neonatal PFC of male and female rats (Willing & Wagner, 2016), mPRβ in the cerebral cortex of adult female rats (Zuloaga et al., 2012), mPRα in the cerebral cortex of adult male and female mice (Meffre et al., 2013), and the expression of PR and mPR α, β, γ, δ and ε in the adult human cerebral cortex (Pang et al., 2013). Concerning E receptors, our data in prepubertal mice also confirm and expand previous data in other species, ages, sexes and brain regions, including the presence of ERβ in the cerebral cortex of peripubertal and adult female rats (Blurton‐Jones & Tuszynski, 2002; Clemens et al., 2019; Drzewiecki et al., 2021) or a comparative study of ERα and β distribution in the cerebral cortex of male and female adult rats (Kritzer, 2002). In addition to ERα and β, GPER has also been found in the PFC of adult mice and rats (Zang et al., 2020).

As mentioned earlier, the PFC undergoes a remarkable anatomical and functional maturation from the prepubertal stage to adolescence (Caballero et al., 2014; Chini & Hanganu‐Opatz, 2021; Delevich et al., 2021; García‐Segura, 2009; Schulz et al., 2016); furthermore, there is evidence that this maturation differs between males and females (De Bellis et al., 2001; Koss et al., 2014, 2015; Premachandran et al., 2020) and that the gonadal hormones P and E play a decisive role, because prepubertal ovariectomy or the exogenous administration of these hormones alters this maturation (Koss et al., 2015; Piekarski, Boivin et al., 2017). Among other things, P and E appear to be necessary to increase the GABAergic transmission onto PYR cells during the transition from the prepubertal stage to adolescence in PFC (Piekarski, Boivin et al., 2017). This increase in GABAergic transmission is necessary for the development of high‐frequency oscillations in PFC circuits, which, in turn, would be the basis for the emergence of new cognitive abilities during adolescence (Chini & Hanganu‐Opatz, 2021; Pöpplau et al., 2024). Concerning the modulation of GABAergic transmission by P and its receptors, previous data come from brain regions other than PFC, such as the hippocampus or cultured cells. Thus, for example, it is known that P and its derivatives promote the phosphorylation of the GABA_A_Rs, which allows, in turn, the accumulation of the receptor in the plasma membrane and thus increases the tonic GABA_A_R‐mediated current in hippocampal neurons (Abramian et al., 2014; Adams et al., 2015; Modgil et al., 2017). In a subsequent study, these researchers found that selective activation of mPRs leads to the phosphorylation of GABA_A_Rs through the activation of PKA and PKC in adult male mice (Parakala et al., 2019). Our present results agree and expand this study by showing that activation of mPRs also increases tonic GABA_A_R‐mediated current and does so by the same mechanism (i.e., PKA/C activation) in PFC layer 2/3 PYR cells of prepubertal male mice. Although we do not know why mPRs modulate tonic current only in males, we think that there might be a different activation of the various mPR subtypes between males and females, which could lead to a different physiological response. In this sense, it is known that mPRα, mPRβ and mPRγ are coupled to an inhibitory G protein (G_i_), leading to the inhibition of the PKA, whereas mPRδ and mPRε activate a stimulatory G protein (G_s_), leading to the opposite effects, the activation of PKA (Pang et al., 2013; Thomas & Pang, 2012). Although we have detected the five subtypes of mPR transcripts in the PFC of both males and females, our qPCR data show that the expression levels of mPRγ (coupled to G_i_) are lower with respect to mPRδ and ε (coupled to G_s_) in males. If this also occurs in the cellular distribution of the receptors (i.e., less mPRγ expression than mPRδ and ε in layer 2/3 PYR cells), the net effect of mPR‐mediated signalling could favour the activation of PKA and the subsequent increase in the tonic current that we observe in layer 2/3 PYR cells in males.

Concerning the role of E receptors in modulating GABAergic transmission, our results show that selective activation of GPER produces an increase in the frequency of IPSCs recorded in PFC layer 2/3 PYR cells in males. Unlike the postsynaptic mechanism proposed for the modulation exerted by mPRs on tonic GABA_A_R‐mediated current (i.e., accumulation of phosphorylated GABA_A_Rs in the postsynaptic cell membrane), the increase in the frequency of IPSCs in response to GPER activation seems to be attributable to a presynaptic mechanism (i.e., an increase in inhibitory discharge on layer 2/3 PYR cells). There is prior evidence that E can quickly modulate phasic GABAergic transmission in different regions of the adult female brain, including the amygdala (Tian et al., 2013), barrel cortex (Clemens et al., 2019) and hippocampus (Hernández‐Vivanco et al., 2022; Mukherjee et al., 2017). There is a discrepancy, however, about the E receptor(s) involved in this modulation. Thus, selective activation of GPER enhanced the frequency of IPSCs recorded in PYR cells of the amygdala (Tian et al., 2013), the activation of ERβ increased the firing rate of parvalbumin‐positive interneurons in the barrel cortex (Clemens et al., 2019), and activation of ERα and/or ERβ decreased the amplitude of IPSCs in the hippocampus (Mukherjee et al., 2017). Our present results in PFC agree with those observed in the amygdala (Tin et al., 2013) and also demonstrate that the increase in the frequency of IPSCs elicited by GPER activation depends on PKA and PKC activation, which, in turn, agrees with previous evidence on how PKA and PKC activation rapidly modulates inhibitory synaptic transmission (Hernández‐Vivanco et al., 2023; Nakamura et al., 2015; Poisbeau et al., 1999). We do not know why the modulation exerted by GPER we found in PFC neurons occurs only in males; it is known, however, that there are sex differences in the role of GPER in diverse brain functions, including memory (Machado et al., 2024) and sexual behaviour (Dovey & Vasudevan, 2020).

## 5 CONCLUSION

Collectively, our results point to a new mechanism by which P and E can rapidly modulate GABAergic transmission in PFC neurons via the activation of their GPCRs, namely mPRs and GPER. Future studies are required to determine, for example, the influence of mPRs and GPER activation on the synaptic weight of excitatory and inhibitory inputs and the intrinsic passive and active electrophysiological properties in both PYR cells and PFC interneurons and whether this influence differs between males and females, as occurs with the modulation of GABAergic transmission. We have shown that the selective activation of mPRs and GPER increases the tonic and phasic GABAergic currents, respectively, via protein kinase A and C. It also remains to be determined whether these results arise from the receptor activation in the membrane of PYR cells and/or on the membrane of interneurons, modifying in this latter neuronal population the passive and active membrane properties and GABA release. Finally, it would be interesting to evaluate the impact of modulation of mPRs and GPER at the cognitive and emotional levels in the PFC. This could open the possibility of using selective mPRs and GPER agonists (e.g., ORG and G1, respectively) as therapeutic agents to treat certain PFC‐related disorders that emerge during the peripubertal stage associated with imbalances in the hormonal milieu within the brain.

## AUTHOR CONTRIBUTIONS

H.T.‐T. and J.G.Y. conceived and designed the study. A.V.‐S., H.T.‐T. and J.G.Y. performed the experiments and participated in the interpretation of data. A.V.‐S., H.T.‐T. and J.G.Y. designed the figures. J.G.Y. wrote the paper with contributions from A.V.‐S. and H.T.‐T. All the authors approved the final version of the manuscript and agree to be accountable for all aspects of the work in ensuring that questions related to the accuracy or integrity of any part of the work are appropriately investigated and resolved. All persons designated as authors qualify for authorship, and all those who qualify for authorship are listed.

## CONFLICT OF INTEREST

None declared.

## Supporting information




**Table S1**. Primers used for qPCR analysis of the different mRNAs of progesterone and oestrogen receptors in prefrontal cortex of female and male mice. Abbreviations: FW, forward primer; RV, reverse primer; Tm, melting temperature.

## Data Availability

Raw data, including recordings, will be provided upon request.
