# Supplementary material for: Membrane progesterone and oestrogen receptors modulate GABAergic transmission in the prefrontal cortex of prepubertal male, but not female, mice
Source: Exp Physiol. 2025 May 1;110(6):888–98. doi: 10.1113/EP092439 (PMC12128468; doi:10.1113/EP092439)
Supplement: Supplementary file 1 — Table S1. Primers used for qPCR analysis of the different mRNAs of progesterone and oestrogen receptors in prefrontal cortex of female and male mice. Abbreviations: FW, forward primer; RV, reverse primer; Tm, melting temperature. [file EPH-110-888-s001.docx]

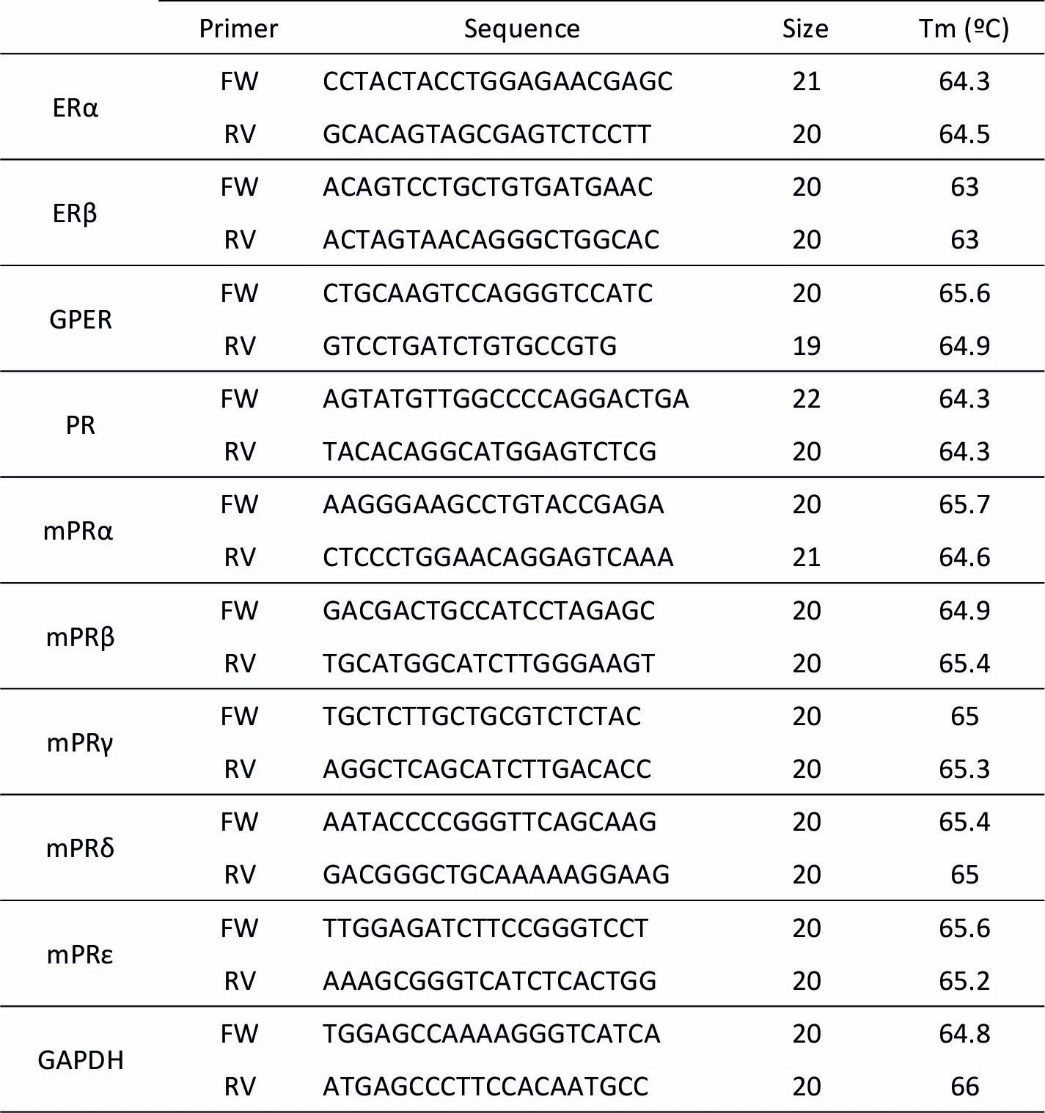


**Supplementary table 1.** Primers used for qPCR analysis of the different mRNAs of progesterone and oestrogen receptors in PFC of female and male mice. FW: forward primer. RV: reverse primer. Tm: melting temperature.
